# Efficacy of Bisphosphonate-Conjugated Sitafloxacin in a Murine Model of *S. aureus* Osteomyelitis: Evidence of “Target & Release” Kinetics and Killing of Bacteria Within Canaliculi

**DOI:** 10.3389/fcimb.2022.910970

**Published:** 2022-06-24

**Authors:** Youliang Ren, Thomas Xue, Joshua Rainbolt, Karen L. de Mesy Bentley, Chad A. Galloway, Yuting Liu, Philip Cherian, Jeffrey Neighbors, Marloes I. Hofstee, Frank H. Ebetino, Thomas Fintan Moriarty, Shuting Sun, Edward M. Schwarz, Chao Xie

**Affiliations:** ^1^ Center for Musculoskeletal Research, University of Rochester Medical Center, Rochester, NY, United States; ^2^ Department of Orthopaedics and Rehabilitation, University of Rochester Medical Center, Rochester, NY, United States; ^3^ Department of Pathology, University of Rochester Medical Center, Rochester, NY, United States; ^4^ Center for Advanced Research Technologies, University of Rochester Medical Center, Rochester, NY, United States; ^5^ BioVinc LLC, Pasadena, CA, United States; ^6^ Department of Pharmacology, Pennsylvania State University, Hershey, PA, United States; ^7^ AO Research Institute Davos, Davos Platz, Switzerland; ^8^ Department of Chemistry, University of Rochester, Rochester, NY, United States

**Keywords:** osteomyelitis, *Staphylococcus aureus*, transmission electron microscopy, antibiotic, bisphosphonate

## Abstract

*S. aureus* infection of bone is difficult to eradicate due to its ability to colonize the osteocyte-lacuno-canalicular network (OLCN), rendering it resistant to standard-of-care (SOC) antibiotics. To overcome this, we proposed two bone-targeted bisphosphonate-conjugated antibiotics (BCA): bisphosphonate-conjugated sitafloxacin (BCS) and hydroxybisphosphonate-conjugate sitafloxacin (HBCS). Initial studies demonstrated that the BCA kills *S. aureus in vitro*. Here we demonstrate the *in vivo* efficacy of BCS and HBCS versus bisphosphonate, sitafloxacin, and vancomycin in mice with implant-associated osteomyelitis. Longitudinal bioluminescent imaging (BLI) confirmed the hypothesized “target and release”-type kinetics of BCS and HBCS. Micro-CT of the infected tibiae demonstrated that HBCS significantly inhibited peri-implant osteolysis versus placebo and free sitafloxacin (p < 0.05), which was not seen with the corresponding non-antibiotic-conjugated bisphosphonate control. TRAP-stained histology confirmed that HBCS significantly reduced peri-implant osteoclast numbers versus placebo and free sitafloxacin controls (p < 0.05). To confirm *S. aureus* killing, we compared the morphology of *S. aureus* autolysis within *in vitro* biofilm and infected tibiae *via* transmission electron microscopy (TEM). Live bacteria *in vitro* and *in vivo* presented as dense cocci ~1 μm in diameter. *In vitro* evidence of autolysis presented remnant cell walls of dead bacteria or “ghosts” and degenerating (non-dense) bacteria. These features of autolyzed bacteria were also present among the colonizing *S. aureus* within OLCN of infected tibiae from placebo-, vancomycin-, and sitafloxacin-treated mice, similar to placebo. However, most of the bacteria within OLCN of infected tibiae from BCA-treated mice were less dense and contained small vacuoles and holes >100 nm. Histomorphometry of the bacteria within the OLCN demonstrated that BCA significantly increased their diameter versus placebo and free antibiotic controls (p < 0.05). As these abnormal features are consistent with antibiotic-induced vacuolization, bacterial swelling, and necrotic phenotype, we interpret these findings to be the initial evidence of BCA-induced killing of *S. aureus* within the OLCN of infected bone. Collectively, these results support the bone targeting strategy of BCA to overcome the biodistribution limits of SOC antibiotics and warrant future studies to confirm the novel TEM phenotypes of bacteria within OLCN of *S. aureus*-infected bone of animals treated with BCS and HBCS.

## Introduction

*Staphylococcus aureus* (*S. aureus*) osteomyelitis is a life-threatening condition, and the standard-of-care (SOC) surgery with antibiotic therapy only results in a ~40% of cure rate (Morgenstern et al., 2021). Recently, it has been found that *S. aureus* can invade and colonize the osteocyte-lacuno canalicular network (OLCN) of cortical bone in mice and patients with chronic osteomyelitis (de Mesy Bentley et al., 2017; de Mesy Bentley et al., 2018). As a result, these bacteria become physically separated from host phagocytes that are too large to enter the OLCN (de Mesy Bentley et al., 2017; Masters et al., 2019; Masters et al., 2022). Additionally, it has been shown that the combination of high-dose local and systemic antibiotic therapies cannot achieve a minimum effective concentration (MEC) in the OLCN compartment, likely due to biodistribution limits, which renders susceptible strains of *S. aureus* resistant to SOC antibiotics (Schwarz et al., 2021).

To overcome the biodistribution limits of SOC treatments, we developed bisphosphonate-conjugated antibiotics (BCA) using a “target and release” approach to deliver antibiotics to bone infection sites (Adjei-Sowah et al., 2021), the theory being that non-nitrogen-containing bisphosphonates, which have a high affinity to hydroxyapatite at eroded bone surfaces but little or no effects on bone cells, could be conjugated to SOC antibiotics *via* a semi-labile, serum-stable, chemical linker, which would specifically target the drug to the bone–bacteria interface and kill the bacteria following acid and/or enzymatic cleavage of the linker releasing an active antibiotic (Adjei-Sowah et al., 2021) in that compartment. As proof of concept, we demonstrated that systemic administration of fluorescent bisphosphonate labels the bone surface adjacent to bacteria in a murine model of *S. aureus* osteomyelitis (Adjei-Sowah et al., 2021).

In selecting candidate SOC antibiotics to conjugate with a bisphosphonate, we completed a comprehensive screen of known regulatory agency-approved drugs for the killing of *S. aureus* biofilm bacteria and small colonies, which are believed to be the bacteria within the OLCN of infected bone. The results showed that sitafloxacin was the most effective bactericidal drug against both methicillin-sensitive *S. aureus* (MSSA) and methicillin-resistant *S. aureus* (MRSA) *in vitro* and *in vivo* (Trombetta et al., 2018). We also demonstrate the ability of bisphosphonate-conjugated sitafloxacin (BCS) and hydroxybisphosphonate-conjugate sitafloxacin (HBCS) to kill *S. aureus in vitro*. Based on this success, we aimed to demonstrate the *in vivo* efficacy of such BCA bone-targeted (and released) antibiotics (including BCS and HBCS) versus placebo, sitafloxacin, and vancomycin in a murine model of implant-associated osteomyelitis *via* longitudinal bioluminescent imaging (BLI), micro-CT, and histology. As the phenotype of antibiotic-killed *S. aureus* within the OLCN has yet to be described, we also used transmission electron microscopy (TEM) of MRSA-infected bone to characterize BCA effects on the bacteria size and morphology.

## Materials and Methods

### *S. aureus* Strains and *In Vitro* Culture

The most prevalent community-acquired MRSA strain of USA300LAC was chromosomally transduced with the bacterial luciferase gene lux to generate USA300 LAC::Lux in an *in vivo* imaging study (Thurlow et al., 2011). The USA300 LAC::Lux was cultured in tryptic soy broth (TSB) media at 37°C overnight as previously described (Kourbatova et al., 2005; Adjei-Sowah et al., 2021). The bioluminescent construct is stably integrated into the USA300 LAC::lux bacterial chromosome and constitutively emits a blue-green light with a maximal emission wavelength of 490 nm only in these live and metabolically active bacteria (Guo et al., 2013).

### Animal Surgeries and Antibiotic Treatments

All *in vivo* experiments with mice were performed following protocols approved by the University of Rochester Committee on Animal Resources (UCAR 2019-015). The surgical approach was performed as previously described (Li et al., 2008; Li et al., 2010; Nishitani et al., 2015). Briefly, a flat stainless steel wire (cross section 0.2 mm × 0.5 mm; MicroDyne Technologies, Plainville, CT, USA) was contaminated with 10^5^ CFU of USA300 LAC::Lux from an overnight culture and surgically implanted through the tibia of 8-week-old Balb/c female mice (n = 4) (Jackson Labs, Bar Harbor, ME, USA). Immediately after surgery, the mice received an intraperitoneal injection of the indicated drug (100 µl/injection) on days 0, 3, 6, 9, and 12 postinfection, or on days 0, 2, 4, 6, 8, 10, 12, and 14 postinfection, except for vancomycin (twice/day). The treatment groups were divided as 1) saline (placebo), 2) vancomycin (110 mg/kg per dose, bid) (Hospira Inc., Lake Forrest, IL), 3) sitafloxacin hydrate 2.5 mg/kg per dose (AChemBlock, Hayward, CA), 4) BCS 5 mg/kg per dose (BioVinc LLC, Pasadena, CA), and 5) HBCS 3 mg/kg per dose (BioVinc LLC, Pasadena, CA). BCS and HBCS were synthesized similarly according to procedures as originally reported in Sedghizadeh et al. (J. Med. Chem. 2017, 60, 2326–2343) and Ebetino et al. (US Patent 10865220) (https://patents.google.com/patent/WO2017210611A1/en). The corresponding bisphosphonate used as the bone-targeting components in BCA (HPBP and HPHBP) was synthesized according to procedures as originally reported in Ebetino et al. (US Patent 10865220) (Adjei-Sowah et al., 2021; Ebetino, Dec. 15, 2020).

### Bioluminescence Imaging

*In vivo* bioluminescence imaging (BLI) was performed on the indicated days 3, 5, and 7, and the data for each mouse were collected continuously. Prior to performing the BLI, the mice (n = 4) were anesthetized with Ketamine/Xylazine (100 mg/20 mg/kg) and the whole right low limb with the pin implant was included in the region of interest (ROI) for digital image taking. The total ROI of each mouse was quantified by using the image analysis software program Amira (Thermo Scientific, Waltham, MA, USA). *In vivo* BLI data were presented on a color scale overlapped with a grayscale photograph of the tibia and quantified as total flux (photons/s) within a standardized circular ROI using Living Image software (Caliper) (Xie et al., 2012).

### Radiographic and Micro-CT

Longitudinal osteolysis was assessed radiographically using a Faxitron Cabinet X-ray system (Faxitron, Wheeling, IL, USA) on days 0, 4, 7, 11, 14, and 18 postinfection as previously described (Li et al., 2010). Micro-CT was performed as previously described (Zhang et al., 2005; Xie et al., 2012; Chen et al., 2016).

### Histology and Transmission Electron Microscopy

Following micro-CT, the implant-loaded tibiae were dissected to remove the stainless-steel pin, decalcified, and embedded in paraffin. At the mid-point of the tibia on the sagittal cuts, five 5-µm tissue sections were obtained from three levels mounted onto slides. One slide from each level of sections was used for H&E staining to show the infection of the whole tibia, and immediately next two adjacent slides were used for Brown–Brenn staining and TEM imaging. TRAP staining was performed to evaluate the osteoclast activity around MRSA-contaminated implants in the bone. One slide per orientation of cut was stained as previously described ( (de Mesy Jensen and di Sant'Agnese, 1992a)). For TEM imaging, the ROIs within serially sectioned paraffin blocks of infected tibia samples were identified using the Brown–Brenn-stained sections, and adjacent unstained slides were reprocessed for transmission electron microscopy using the “pop-off” technique, as previously described (de Mesy Jensen and di Sant'Agnese, 1992b). Briefly, slides were deparaffinized in three changes of xylene and then rehydrated through a graded series of ethanol back to ddH_2_O. Rehydrated sections on slides were postfixed overnight at 4°C in 0.1 M phosphate-buffered 2.5% glutaraldehyde then 1% OsO_4_ for 20 min at room temperature. The slides were washed in ddH_2_O, dehydrated in a graded series of ethanol to 100% (×3), and infiltrated with a 1:1 mixture of 100% ethanol and Spurr’s resin then 100% Spurr’s resin overnight at room temperature. The ROI (etched with a diamond pen on the backside of the slides) was reidentified after the slides were drained of excess resin. A size 3 BEEM capsule filled with fresh resin was placed over the paraffin section’s ROI and polymerized 24 h at 65°C. The BEEM capsules, with entrapped ROIs, were popped off slides by dipping them for 5–10 s rapidly in liquid nitrogen. The popped-off block was trimmed to the ROI, thin sectioned at ~70 nm, and placed onto formvar carbon-coated nickel slot grids for imaging using a Gatan Erlangshen 11-megapixel digital camera (Pleasanton, CA) and a Hitachi 7650 TEM (Santa Clara, CA).

### Transmission Electron Microscopy of *In Vitro* Model of *Staphylococcus* Abscess Communities

Collagen hydrogels with fully formed *Staphylococcus* abscess communities (SACs) were fixed in 4.0% paraformaldehyde/1.0% glutaraldehyde in 0.1 M Sorensen’s buffer for 48 h, rinsed in buffer, postfixed in buffered 1.0% osmium tetroxide, dehydrated in a graded series of ethanol to 100% (×3), and transitioned into 100% propylene oxide, 1:1 propylene oxide/Epon-Araldite epoxy resin, then 100% epoxy resin overnight. The next day, the hydrogels were embedded flat into the caps of BEEM capsules and polymerized overnight at 60°C, and the caps were placed back on a BEEM capsule filled with 100% resin and polymerized at 60°C. The SACs were sectioned at 1 micron and stained with Toluidine blue to identify a SAC within the hydrogel for subsequent thin sectioning at 70 nm onto formvar/carbon copper slot grids. The grids were stained with uranyl acetate and lead citrate and imaged using a Hitachi 7650 TEM, as stated above.

### Statistical Analysis

Data were analyzed by using GraphPad Prism software 9.0 and are presented with the mean +/- SD. Data were analyzed by a one-way ANOVA and two-way ANOVA. p values of <0.05 were considered significant (* indicates statistically significant difference of p < 0.05; ** indicates p< 0.01).

## Results

### BCA Effects on *In Vivo* MRSA Planktonic Growth

**Figure 1:**
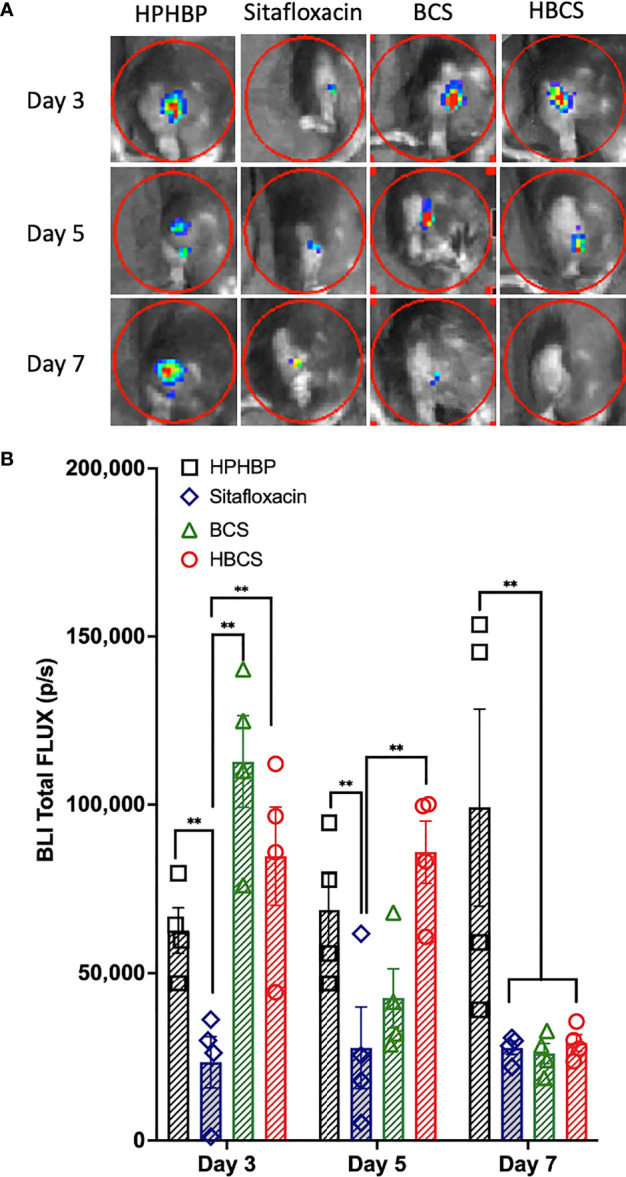
*In vivo* evidence of extended antimicrobial efficacy of bisphosphonate-conjugated sitafloxacin. Mice (n = 4) were challenged with a USA300LAC::Lux-contaminated transtibial pin and were given the indicated drug on days 0, 3, 6, 9, and 12 postinfection. *In vivo* BLI was performed on the indicated days 3, 5, and 7 **(A)**, and the data for each group are shown continuously as mean +/- SD for the group (**p < 0.001 two-way ANOVA) **(B)**. Of note is that the BLI in HPHBP-treated mice (negative control for HBCS) remained elevated throughout infection, while the free sitafloxacin treatment led to an immediate reduction in BLI and maximal inhibition of BLI in both BCS- and HBCS-treated mice was delayed until ~5–7 days postinfection and remained low thereafter.

We have previously shown that the BLI signal from bioluminescent *S. aureus* following transtibial implant infection of mice corresponds to robust planktonic growth of the bacteria and that the signal decreases significantly ~7 days postinfection due to host immunity that prohibits planktonic growth, and the abscess of the detectible BLI signal from the persistent biofilm bacteria (Nishitani et al., 2015). Thus, to assess the effects of BCS and HBCS on *in vivo* MRSA planktonic growth following implant-associated bone infection, we performed longitudinal BLI of mice challenged with a bioluminescent strain of USA300 LAC::Lux treated with BCS, HBCS, bisphosphonate (HPHBP) negative control, and SOC sitafloxacin positive control (**Figures 1A, B**). The results demonstrated that the HPHBP-treated mice had robust BLI activity, while free sitafloxacin decreased BLI throughout the 1-week study period. In contrast, maximal BCS and HBCS inhibition of BLI was delayed until days 5–7 postinfection and extended thereafter, consistent with the predicted “target and release” kinetics of the conjugate binding at the bone–bacteria interface, and subsequent linker cleavage to release the active antibiotic.

### BCA Effects on Peri-Implant Osteolysis and Osteoclasts

**Figure 2:**
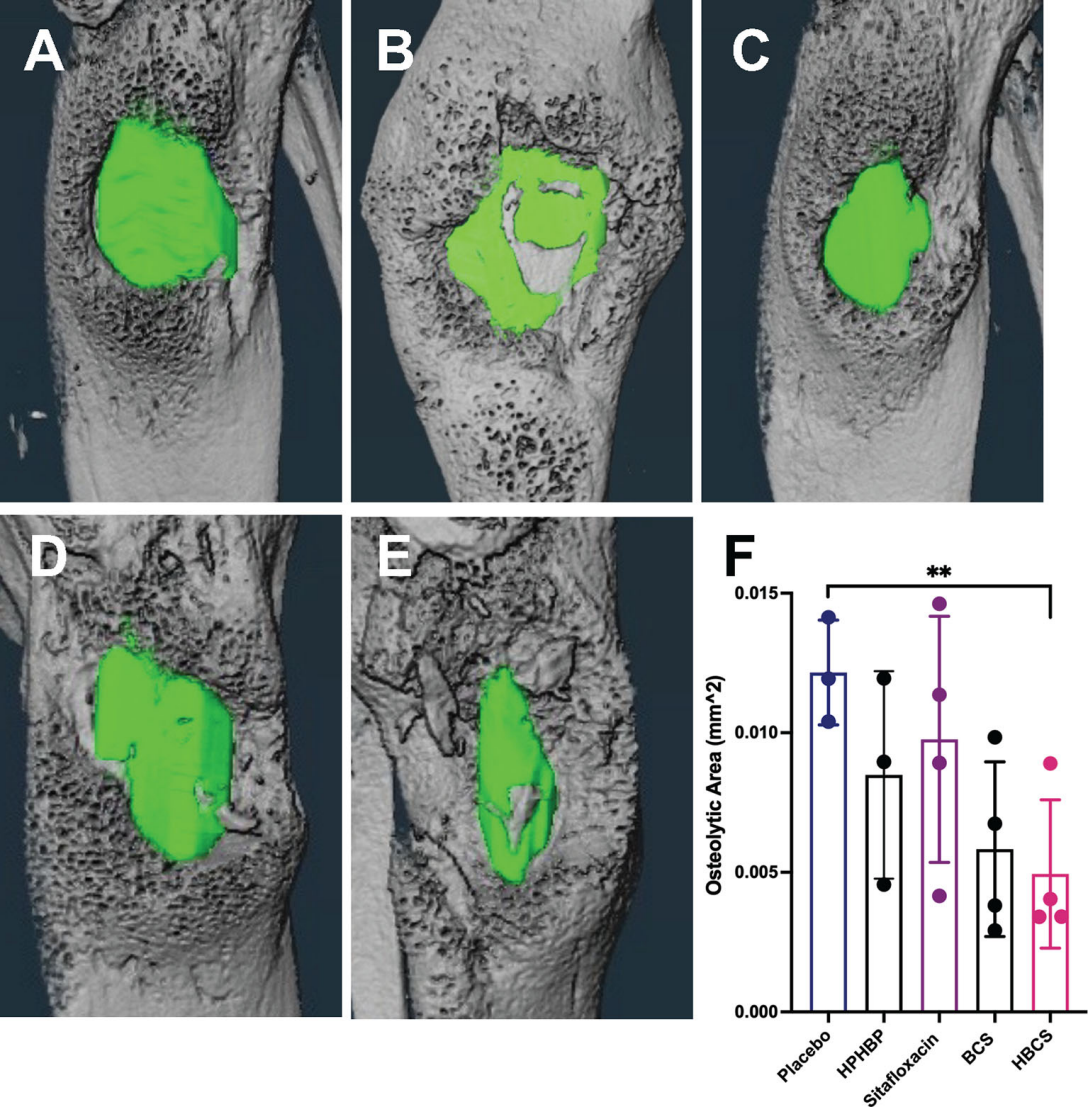
*In vivo* evidence of reduced peri-implant osteolysis in HBCS- and BCS-treated mice vs. free sitafloxacin and controls. Mice (n = 4) were challenged with a USA300LAC::Lux contaminated transtibial pin and were given the indicated drug on days 0, 2, 4, 6, 8, 10, 12, and 14 postinfection. The infected tibiae were harvested on day 14 and processed for micro-CT to quantify peri-implant osteolysis. Representative 3D lateral view of renderings of infected tibiae from mice treated with placebo **(A)**, HPHBP **(B)**, sitafloxacin **(C)**, BCS **(D)**, and HBCS **(E)** are shown with the osteolysis area (green). The osteolysis area for each tibia is presented with the mean +/- SD for each group **(F)** **p < 0.01 one-way ANOVA).

**Figure 3:**
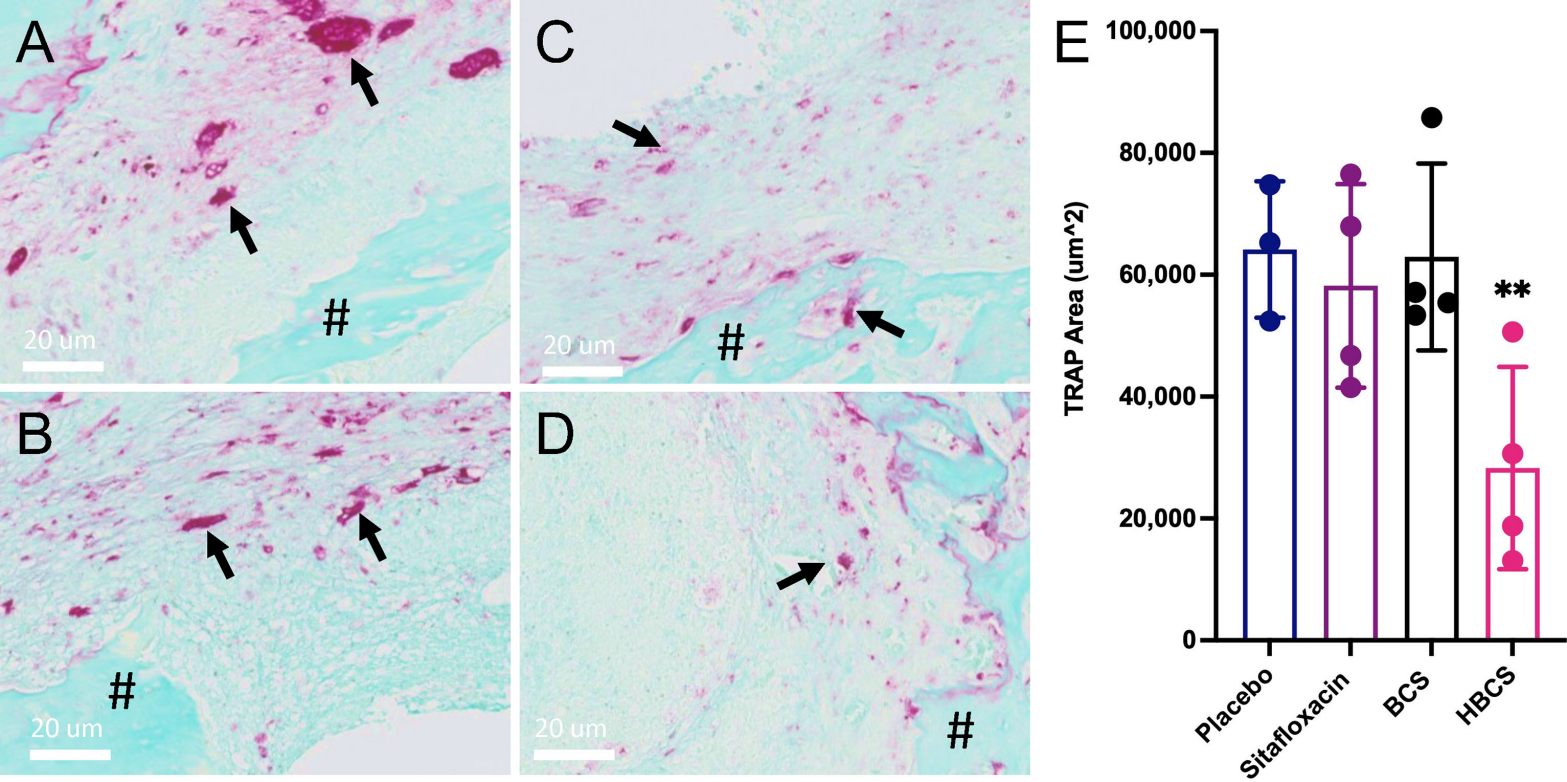
HBCS treatment reduces osteoclast numbers in mice with implant-associated osteomyelitis. The infected tibiae (n = 4) described in **Figure 2** were processed for TRAP stained histology, and semiautomated histomorphometry was performed to quantify the TRAP+ area. Representative high-resolution (×40) images of placebo **(A)**, sitafloxacin **(B)**, BCS **(C)**, and HBCS **(D)** are shown with quantification of the TRAP-stained area **(E)**, in which the data for each tibia are presented with the mean +/-SD for the group (**p < 0.05 one-way ANOVA). All black arrows in **(A–D)** indicate the osteoclast and # mean the presence of sequestrum in the medullary canal. The TRAP+ area for HPBP-treated mice was similar to placebo (data not shown).

To assess the effects of BCA on MRSA-induced bone resorption around the septic pins, we performed micro-CT analyses and TRAP-stained histology on the infected tibiae. Micro-CT analysis of peri-implant osteolysis revealed that only HBCS (**Figure 2E**) significantly inhibits bone loss and with a statistic difference (p < 0.05) (**Figure 2F**). Consistent with the observed peri-implant osteolysis (**Figure 3**), large numbers of TRAP+ osteoclasts were observed around MRSA-contaminated pins from mice treated with placebo, sitafloxacin, and the non-nitrogen-containing bisphosphonate controls (**Figures 3A–C**). Interestingly, while HBCS treatment (**Figure 3D**) significantly reduced osteoclast numbers (**Figure 3E**) as expected, BCS did not, which could reflect the higher concentrations of this drug localized at that infected site and thus greater efficacy, but this remains an unexplained observation for future study.

### BCA Effects on MRSA Within OLCN

**Figure 4:**
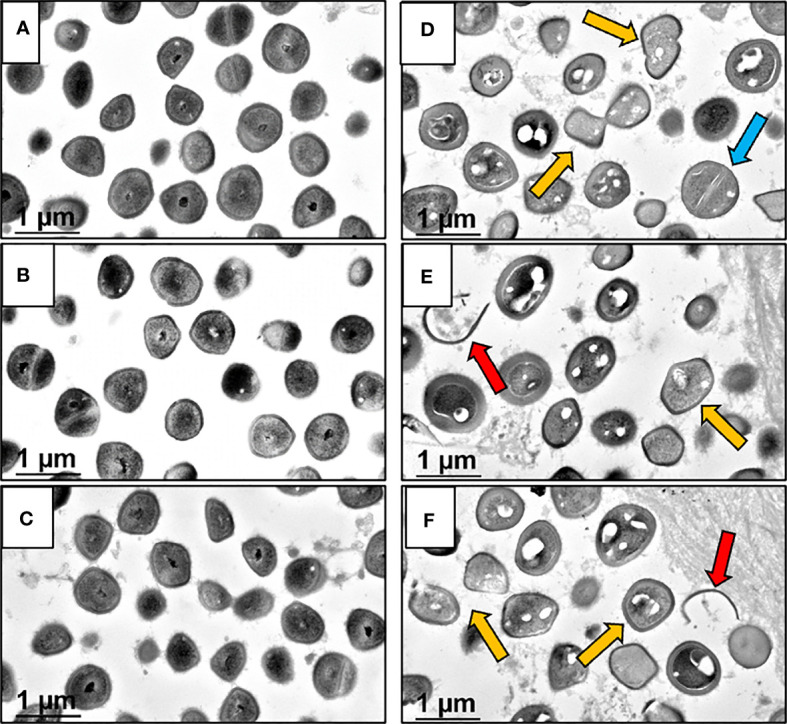
Morphology of *S. aureus* within untreated and sitafloxacin-treated *in vitro* SACs analyzed by TEM. *In vitro* Staphylococcal abscess communities (SAC) grown in collagen hydrogels were processed for TEM imaging to illustrate the morphologic features of 100% viable untreated bacteria with dense black interiors **(A–C)** ×30,000, compared to sitafloxacin-treated SACs **(D–F)** ×30,000, displaying vacuolated degenerating (gold arrows) and dead bacteria (ghosts, red arrows). Note in **(D)**, a single viable *S. aureus* (blue arrow) non-vacuolated coccus with an intact septal wall compared to the majority of vacuolated dying bacteria.

**Figure 5:**
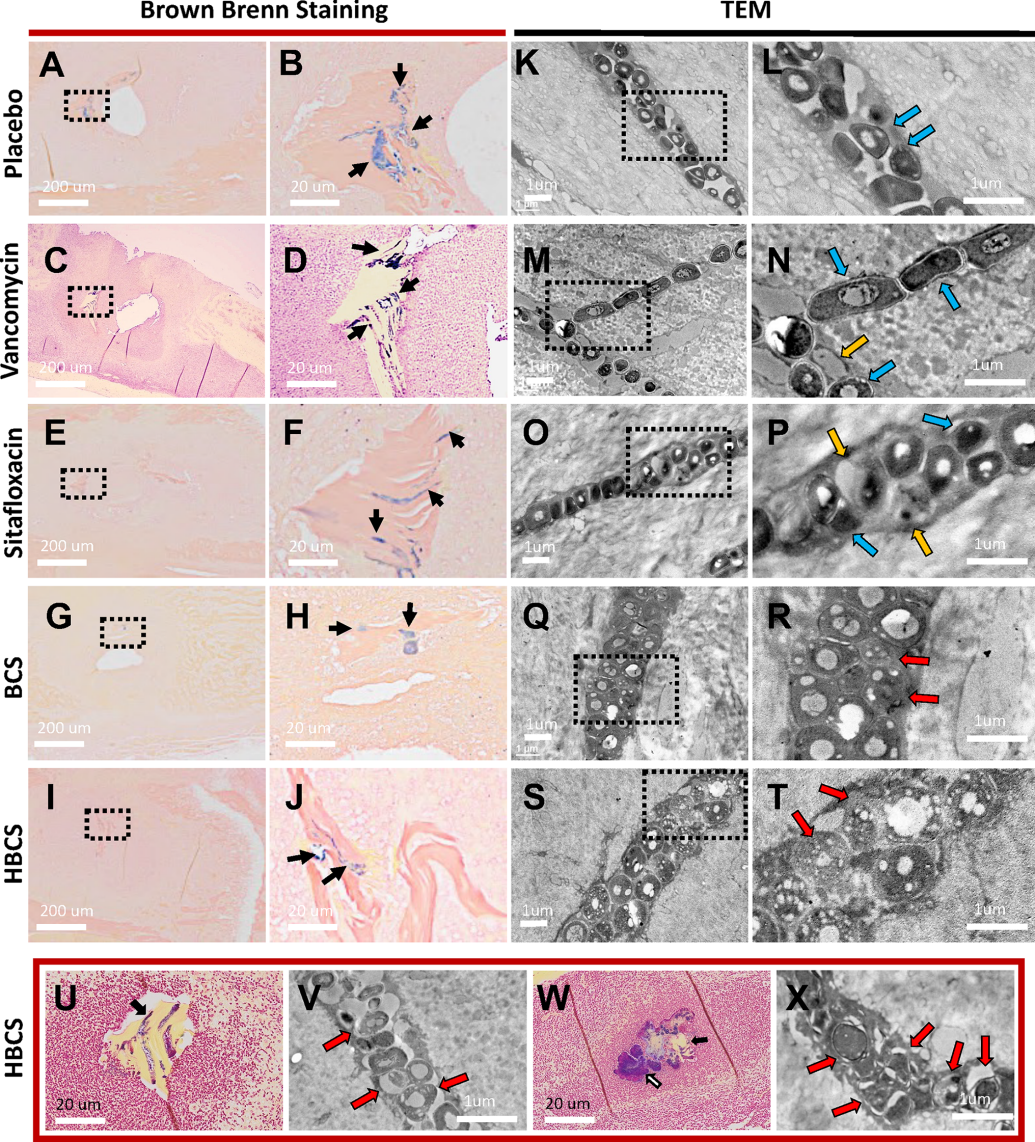
TEM evidence of HBCS and BCS killing of MRSA within canaliculi. Histology from the infected tibiae described in **Figure 3** were processed for Brown and Brenn staining **(A–J, U, W**) and subsequent TEM **(K–T, V, X)**. Representative low-power (×0.5) images containing necrotic bone fragments with Gram-positive bacteria in the marrow space **(A, C, E, G, I)**, with high-power (×5) images of the dashed boxed region of interest **(B, D, F, H, J)** are shown to illustrate the extent of biofilm formation (black arrows) in the indicated treatment groups on day 14 postinfection. Representative TEM images of the bacteria within canaliculi of the infected bone are shown at low power (×12,000, **K, M, O, Q, S**), with high-power images (×25,000) of the dashed boxed region of interest **(L, N, P, R, T)** shown to illustrate the morphology of the bacteria within canaliculi on day 14 postinfection. Note that most bacteria in the placebo and vancomycin- and sitafloxacin-treated tibiae are dense (dark), and there are a few ghosts (red arrows) and degenerating bacteria (yellow arrows). In contrast, bacteria in BCS- and HBCS-treated tibiae are less dense and contain small vacuoles and holes >100 nm. To see if the morphology of these vacuole-containing dying bacteria with hole >100 nm changes to a more necrotic phenotype, we repeated the HBCS treatment of mice with an MRSA-infected transtibial pin for 14 days, left these mice untreated for another 14 days **(U, V)**, or throughout all 28 days of treatment **(W, X)**, then harvested the infected tibiae 28 days post-op for histology and TEM. A representative Brown and Brenn-stained section containing a necrotic bone fragment with Gram-positive bacteria is shown at ×10 **(U, W)** with a high-power (×15,000) TEM of the bacteria in the canaliculus **(V, X)**. Note that the large number of ghosts and necrotic bacteria had lost their semirigid structure, several ruptures and displacement of membranes, lysis, and extrusion of the intracellular content with large holes (red arrows) in HBCS treatment groups.

**Figure 6:**
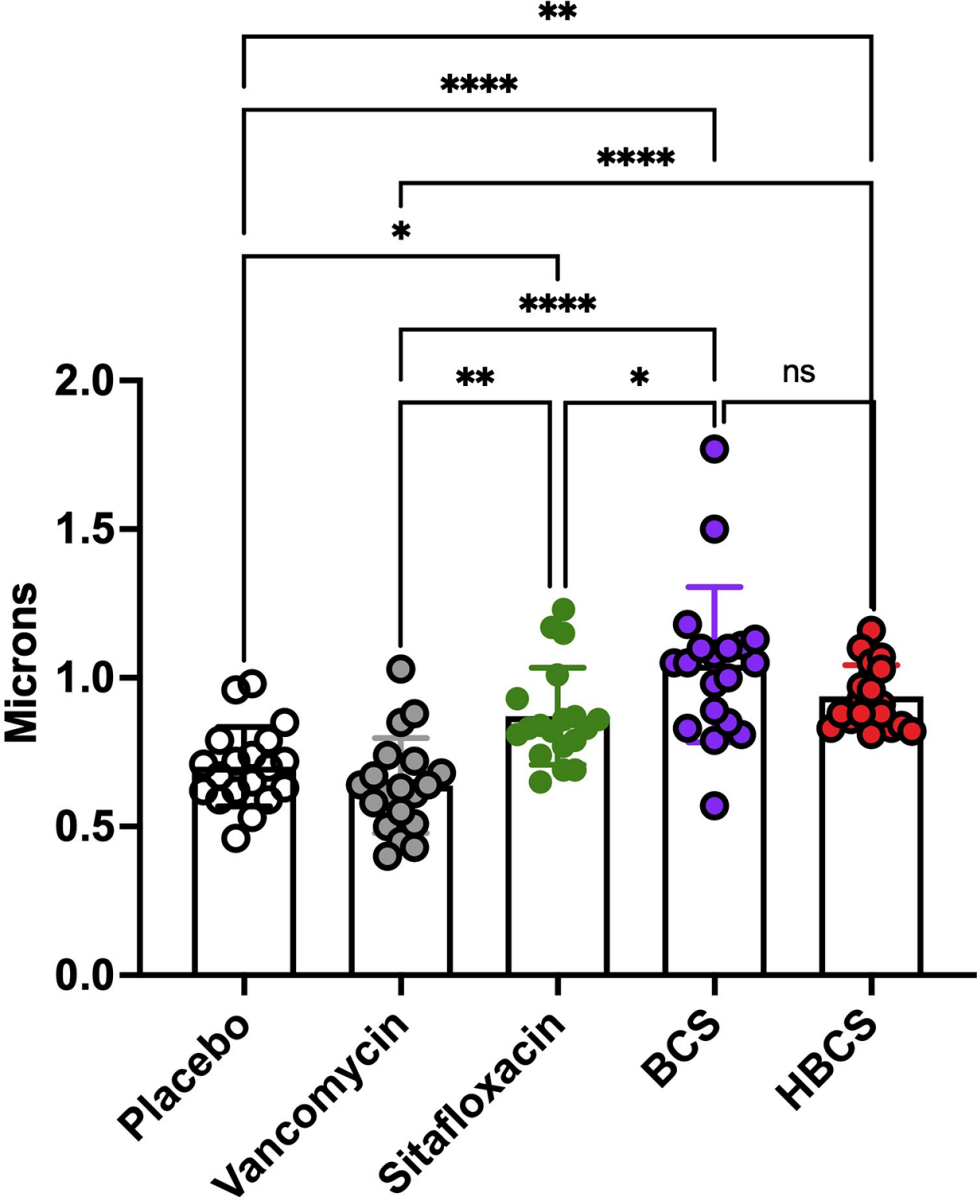
HBCS and BCS treatment of MRSA-infected mice causes swelling of bacteria within canaliculi. The TEM images described in **Figure 5** were used to quantify the diameter of the bacteria in the canaliculi of mice given the indicated treatment for 14 days following infection. The diameter of each bacterium (n = 4) is presented with the mean +/-SD for the group (two-way ANOVA: ns: no statistical significant, ****p < 0.0001, **p < 0.005, *p < 0.05).

At the end of demonstrating that BCA kills MRSA within OLCN of infected bone *via* TEM assessment of necrotic bacteria morphology, we interrogated TEM phenotypes of *S. aureus* within *in vitro* biofilm, as previously described (Hofstee et al., 2021). By TEM imaging of SACs grown in a collagen hydrogel (**Figures 4A–C** (low magnification), **Figures 4D–F** (high magnification)), we confirmed the phenotype of vital *S. aureus in vitro*. Here, *S. aureus* appeared as dense cocci and diplococci undergoing binary fission. In comparison, sitafloxacin-treated SAC bacteria displayed cell wall remnants of dead bacteria (red arrows) and degenerating vacuolated bacteria (gold arrows) with an occasional rare viable bacteria (blue arrow) (**Figures 4D–F**). With these phenotypes identified, we performed TEM on Gram+ bone fragments from Brown and Brenn-stained histology (de Mesy Bentley et al., 2021) and looked for novel phenotypes consistent with the biology of antibiotic-killed bacteria (i.e., cell swelling with large numbers of vacuoles) from drug-inhibited DNA gyrase and topoisomerase IV (Chen et al., 1996). Consistent with our prior studies, the TEM phenotypes of MRSA in OLCN of vancomycin and sitafloxacin were similar to those of placebo controls (**Figure 5**). Essentially, all of these bacteria had a dense vital phenotype, although a few bacterial “ghosts” and bacteria with an apparent central vacuole were readily observed, which could be from histologic reprocessing artifacts. In contrast, TEM of MRSA-infected tibiae from mice treated with BCS and HBCS for 14 days showed bacteria with a novel phenotype of swollen cocci to include a very large central vacuole (>1 mm) or many small vacuoles (**Figures 5Q–T**). To determine if these novel TEM phenotypes were actually antibiotic-killed bacteria, we repeated the *in vivo* challenge and BCA treatment for 28 days (**Figures 5U–X**) to test the hypothesis that the highly vacuolized bacterial cells would contract to a necrotic phenotype with time. We also performed histomorphometry on the TEM images described in **Figures 5K–T** to quantify the size of the bacteria in the OLCN. The results confirmed that *in vivo* BCS and HBCS treatment significantly increased the size of the bacteria within the OLCN (**Figure 6**).

## Discussion

Based on the fundamental concepts of *S. aureus* bone infection, the standard-of-care treatments for implant-associated osteomyelitis were established in the 1970s. These treatments involve 1) removal of the infected implant, 2) extensive surgical debridement of adjacent bone and soft tissues, and 3) filling of the bone void with antibiotic-loaded acrylic cement. By the mid-1980s, implementation of this standard of care resulted in 5-year success (survival) rates of 77% (Buchholz et al., 1984). Remarkably, data from the 2018 International Consensus Meeting on Musculoskeletal Infections reported no changes in bone infection rates, the primary pathogen, treatment algorithm, and poor outcomes since this original standard of care was established half a century ago (Parvizi et al., 2018; Saeed et al., 2019; Schwarz et al., 2019a). However, basic and translational research in this field has produced several major discoveries that have changed our thinking about microbial pathogenesis, antibiotic resistance, and the treatment of bone infection. Most notable is the discovery of *S. aureus* invasion and colonization of the OLCN during chronic osteomyelitis (de Mesy Bentley et al., 2017; de Mesy Bentley et al., 2018). Based on this observation, there is now broad consensus among key opinion leaders in this field that SOC antibiotic therapies cannot irradicate *S. aureus* bone infection due to biodistribution limits even at the highest doses that can be given systemically and locally (Schwarz et al., 2019b; Schwarz et al., 2021).

To overcome the biodistribution limits of SOC antibiotics toward the eradication of *S. aureus* within OLCN of infected bone, we have been exploring the potential of BCA. This rationale comes from the rich history of safe and effective bisphosphonate therapies, which have proven bone targeting with great specificity and minimal side effects on soft tissues (Sun et al., 2021). Of note is that several different types of bone-targeting drug candidates have been developed by linking FDA-approved drugs to bisphosphonate (e.g., bortezomib (Wang et al., 2018; Wang et al., 2020)), and the subject has been extensively reviewed (Farrell et al., 2018; Xing et al., 2020). Based on this, we aimed to create a bone-targeted antibiotic conjugate that could achieve sustained concentrations of a drug well above the MEC at the site of bone infection. In support of this concept, we recently demonstrated that systemic administration of a BP-conjugated fluorophore (AF647-ZOL) specifically labels the cortical surface of the bone in immediate proximity to *S. aureus* bacteria during chronic osteomyelitis (Adjei-Sowah et al., 2021). We were also encouraged by our initial success with BP-conjugated ciprofloxacin for the oral indication (Sedghizadeh et al., 2017). However, since fluoroquinolones suffer from reduced activity against established biofilms, which is particularly true for Gram-positive species such as *S. aureus* (Amorena et al., 1999; Melchior et al., 2006), we developed BP–sitafloxacin conjugates on the basis of our prior regulatory agency-approved drug library screen for bactericidal activity against *S. aureus* SCV (Trombetta et al., 2018; Trombetta et al., 2019). As initial studies demonstrated the efficacy of BCS and HBCS to kill MSSA and MRSA within *in vitro* biofilm (Adjei-Sowah et al., 2021), here we aimed to evaluate their efficacy *in vivo* versus SOC antibiotics and non-antibiotic-conjugated bisphosphonate controls.

Here we utilized *in vivo* bioluminescent imaging of *S. aureus* planktonic growth during the establishment of implant-associated osteomyelitis (Nishitani et al., 2015), as an initial proof of concept of BCA “target and release” efficacy in mice. As we hypothesized, a similar decrease in the BLI signal was achieved with both BCS and HBCS versus parent sitafloxacin, but the maximal decrease was delayed ~5 days and extended beyond that time, which is consistent with the pharmacokinetics of BCA binding to the bone surface and subsequent cleavage of the linker to release sitafloxacin (**Figure 1**). Another critical proof of concept is our demonstration that HBCS significantly inhibits infection-induced peri-implant osteolysis compared to the sitafloxacin and non-nitrogen-containing bisphosphonate parent components (**Figure 2**). This efficacy is hypothesized to be from the BCA killing of *S. aureus* biofilm bacteria within the OLCN, which is not achieved by sitafloxacin due to the aforementioned biodistribution limits. Additionally, since the nitrogen-containing bisphosphonates have great potency to inhibit osteoclasts, the bisphosphonates used as the bone-targeting component of BCA are designed to be innocuous or have minimal effects on bone cells (Adjei-Sowah et al., 2021), and the lack of efficacy of our “non-nitrogen-containing” bisphosphonate controls (HPHBP and HPBP) is consistent with our design and hypothesis. However, one unexplained finding in our study is that only HBCS reduces osteoclast number, and not BCS (**Figure 3**). While we can speculate on the reasons for this, it is clear that future studies are needed to address this issue.

Another critical proof of concept that is needed to move BCA toward clinical translation is the demonstration that they kill *S. aureus* within the OLCN of infected bone *in vivo* (**Figures 4**). However, this evidence comes with several challenges including the absence of validated outcome measures. To this end, here we aimed to identify unique TEM ultrastructural phenotypes of MRSA within OLCN of infected bone from mice treated with BCA versus controls. While we indeed found some unique phenotypes (**Figures 5**, **6**) which are consistent with literature reports of killed bacterial cell swelling with large numbers of vacuoles from drug-inhibited DNA gyrase and topoisomerase IV (Chen et al., 1996), several validation studies will be needed to more firmly conclude that BCA kills *S. aureus* within OLCN. Nonetheless, these positive findings support further preclinical and clinical research of BCA as an adjuvant therapy to treat chronic osteomyelitis.

## Conclusions

Here we describe the initial *in vivo* “target and release” pharmacokinetics of bisphosphonate-conjugated antibiotics and evidence that these compounds kill MRSA within the OLCN of infected bone. If these results can be substantiated by large preclinical studies, clinical trials to develop these novel drugs for osteomyelitis could be warranted.

## Data Availability Statement

The raw data supporting the conclusions of this article will be made available by the authors, without undue reservation.

## Ethics Statement

The animal study was reviewed and approved by the University of Rochester Committee on Animal Resources (UCAR 2019-015). Written informed consent was obtained from the owners for the participation of their animals in this study.

## Author Contributions

Conceptualization, CX, ES, FE, and SS; data curation, YR, TX, JR, KB, CG, YL, PC, JN, MH, FE, TM, SS, ES, and CX; formal analysis, YR, TX, JR, KB, CG, YL, PC, JN, MH, FE, TM, SS, ES, and CX; funding acquisition, CX, ES, FE, and SS; investigation, YR, TX, JR, KB, CG, YL, PC, JN, MH, FE, TM, SS, ES, and CX; methodology, YR, TX, JR, KB, CG, YL, PC, JN, MH, FE, TM, SS, ES, and CX; project administration, CX, ES, FE, SS; supervision, CX, ES, FE, SS; validation, YR, KB, FE, SS, ES, and CX; writing—original draft, YR, TX, JR, KB, CG, YL, PC, JN, MH, FE, TM, SS, ES, and CX; writing—review and editing, CX, ES, FE, SS. All authors contributed to the article and approved the submitted version.

## Funding

This work was supported by grants from the National Institutes of Health (SBIR R44 AI125060, NIAMS P50 AR072000 and NIAMS P30 AR069655).

## Conflict of Interest

Authors PC, JN, FE and SS were employed by company BioVinc LLC. SS and FE hold equity in BioVinc LLC (Pasadena, CA) which partially sponsored this research. PC, SS, and FE are inventors on patents related to this work.

The remaining authors declare that the research was conducted in the absence of any commercial or financial relationships that could be construed as a potential conflict of interest.

## Publisher’s Note

All claims expressed in this article are solely those of the authors and do not necessarily represent those of their affiliated organizations, or those of the publisher, the editors and the reviewers. Any product that may be evaluated in this article, or claim that may be made by its manufacturer, is not guaranteed or endorsed by the publisher.
